# A Review of *Chenopodium quinoa* (Willd.) Diseases—An Updated Perspective

**DOI:** 10.3390/plants10061228

**Published:** 2021-06-16

**Authors:** Carla Colque-Little, Daniel Buchvaldt Amby, Christian Andreasen

**Affiliations:** Department of Plant and Environmental Sciences, University of Copenhagen, Højbakkegaard Allé 13, DK2630 Taastrup, Denmark; cxl@plen.ku.dk (C.C.-L.); amby@plen.ku.dk (D.B.A.)

**Keywords:** causal agents, downy mildew, pathogenicity, *Peronospora*, resistance factors, severity, quinoa diseases, quinoa disease assessment

## Abstract

The journey of the Andean crop quinoa (*Chenopodium quinoa* Willd.) to unfamiliar environments and the combination of higher temperatures, sudden changes in weather, intense precipitation, and reduced water in the soil has increased the risk of observing new and emerging diseases associated with this crop. Several diseases of quinoa have been reported in the last decade. These include *Ascochyta caulina*, *Cercospora* cf. *chenopodii*, *Colletotrichum nigrum*, *C. truncatum*, and *Pseudomonas syringae*. The taxonomy of other diseases remains unclear or is characterized primarily at the genus level. Symptoms, microscopy, and pathogenicity, supported by molecular tools, constitute accurate plant disease diagnostics in the 21st century. Scientists and farmers will benefit from an update on the phytopathological research regarding a crop that has been neglected for many years. This review aims to compile the existing information and make accurate associations between specific symptoms and causal agents of disease. In addition, we place an emphasis on downy mildew and its phenotyping, as it continues to be the most economically important and studied disease affecting quinoa worldwide. The information herein will allow for the appropriate execution of breeding programs and control measures.

## 1. Introduction

Agriculture is affected by global climate change. Non-traditional crops with high nutritional value and the ability to cope with abiotic stress are of special interest in today’s world. Quinoa (*Chenopodium quinoa* Willd.) is an ancient crop that exhibits remarkable tolerance to frost, salt, and drought. Moreover, it is highly nutritious and has a vast genetic diversity resulting from its fragmented and localized production over the Andean region. The recent introduction and cultivation of quinoa in novel environments has resulted in a wider spectrum and higher intensity of infectious diseases. Oomycetes and fungi are the two most important eukaryotic plant pathogens [1]; their predominance on the quinoa pathobiome is also evident.

Diseases of quinoa have been reviewed previously [2,3,4,5,6]. However, an update is necessary because new emerging diseases of the quinoa mycobiome are being discovered. Taxonomy based on the morphological characteristics and nomenclature of fungi is relatively conserved and informative when high-level classifications (genus level) are considered. However, there is uncertainty when lower-level phylogenies (species level) are considered due to the fast-evolving traits and phenotypic plasticity of fungi [7]. As a result, DNA and molecular sequence-database comparisons techniques have been employed, along with various DNA fingerprinting and more advanced and complex methods such as whole-genome sequencing, for the identification of plant pathogens [8,9].

The universal nuclear ribosomal primers developed by White et al. (1990) for PCR amplification of the internal transcribed spacer (ITS) region have become a key component the description and characterization of fungal diversity [10]. In addition to ITS, various other markers exist for multi-locus sequencing. It is commonly used by combining ITS with other relevant genomic regions (e.g., COX I, calmodulin, and TEF1 gene regions). It has proven helpful and necessary for the accurate identification of microbial plant pathogens [11,12,13]. Such molecular approaches should be paired with pathogenicity assessments, including the description of disease symptoms, isolation and artificial inoculation of quinoa tissue, recording of symptoms, and re-isolation. These tests are known as Koch’s postulates [14,15,16]; their validation discriminates an opportunistic association from a pathogenic-type interaction.

This review aims to provide an updated overview of microbial plant pathogens causing disease in quinoa, focusing on the morphological characterization and molecular identification of the causal agents. Research carried out in the Andean countries some decades ago provides insightful and valuable reports, described herein. We compiled and analyzed existing information, with a marked emphasis on downy mildew.

## 2. Downy Mildew of Quinoa

### 2.1. Nomenclature and Distribution

The oomycete *Peronospora variabilis* Gäum. 1919 [17] is the causal agent for downy mildew on *C. quinoa* (www.indexfungorum.org, accessed on 10 June 2021) and *C. album* L. The genus *Peronospora* belongs to the *Peronosporaceae* family (Peronosporales order), which are highly physiologically specialized, biotrophic organisms. Phytopathogenic oomycetes are eukaryotic microbes with filamentous vegetative growth and spores for reproduction (fungus-like). Molecular analysis revealed they are among the *Stramenopiles* (or heterokont), closely related to golden-brown algae and diatoms [1,18,19,20]. Fundamental features are:

1. Oomycetes cell walls are mostly composed of glucans, in contrast to chitin from fungi [14].
2. Most oomycetes are insensitive to azole fungicides (e.g., ketoconazole) because they do not have the ergosterol pathway needed to activate the azole-fungicide mode of action [21,22,23].
3. During their vegetative state, oomycetes are diploid compared to haploid or dikaryotic fungi [1].

Due to taxonomic confusion, downy mildew was previously classified as *Peronospora farinosa* and considered as such by most studies for about 50 years [24,25,26]. Byford (1967a,b) [27,28] investigated cross-inoculation experiments and concluded the division of three *formae speciales* (f. spp.) Table 1.

Later, a phylogenetic study on *P. variabilis* of *C. quinoa* and *C. album* from different geographical regions showed that both are located in the same phylogenetic cluster with no evidence to separate them into different taxa [29,30,31,32]. Morphological, molecular, and biological host specialization analyses revealed that a narrow species concept is more appropriate for the downy mildews. The available evidence strongly suggests that the host range of *P. variabilis* is limited to *C. quinoa* and *C. album* [29], that of *P. effusa* is limited to spinach [33,34], and more recently that of *P. chenopodii* has been shown to be limited to *C. hybridum* L. (maple leave goosefoot), *P. chenopodii-ambrosioides* to *C. ambrosioides* L. (Jesuit’s tea, Payqu), *P. chenopodii-ficifolii* to *C. ficifolium* Sm. (fig leave goosefoot) [13], *P. chenopodii-polyspermi* to *C. polyspermum* L. (many-seeded goosefoot), and *P*. *schachtii* to sugar beet [26]. In older literature, *P. farinosa* was used as the causal agent of downy mildew of quinoa. However, the species name “*farinosa*” had been ascribed to an unrelated genus (*Atriplex*) and is no longer valid as a species name for *Peronospora* [35].

**Table 1:** *Peronospora* species current identity and classification by Byford [27,28].

Host (Genus/Species)	Pathogen Current Identity	Byford Classification (f. spp.) *P. farinosa formae speciales*
*Beta* spp.	*P. schachtii* [26]	*P. farinosa* f. sp. *betae*
*C. álbum + C. quinoa*	*P. variabilis* [29,30]	*P. farinosa* f. sp. *chenopodii*
*Spinacia* oleracea	*P. effusa* [33,34]	*P. farinosa* f. sp. *spinaciae*

**Figure 1:**
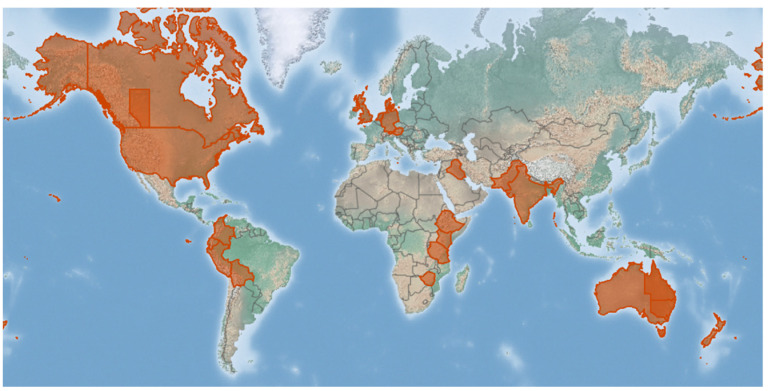
Downy mildew disease of *Chenopodium* spp. distribution map (CAB international, last modified 21 November 2019 via www.cabi.org/isc/datasheet/39704 (accessed on 10 June 2021).

The earliest report of downy mildew infecting quinoa in South America came from Martin Cardenas (1941), who found it infecting quinoa in Cochabamba, Bolivia, and identified it as *P. farinosa* [36]. *P. variabilis* has been documented throughout the world (Figure 1) [26,29,30,31,32,37,38,39,40,41,42,43,44,45,46,47,48,49,50,51,52] wherever quinoa is cultivated. It is expected to become ubiquitous in all quinoa cropping areas as oospores found in seeds have also been seen in old dried leaves [32,53,54]. Moreover, *C. album* (known as goosefoot, fat hen, or lamb’s quarters) [55] is frequently infected by downy mildew throughout Europe because it is conspecific [56] with the *P. variabilis* from *C. quinoa*. Therefore, it is likely to be a reservoir for the pathogen and an alternative host [29,52,56]. Other *Chenopodium* species, such as *C. murale* L. (nettle leaf goosefoot), *C. ambrosoides* L. (Indian goosefoot, Mexican tea), *C. berlandieri* Moq. (pit seed goosefoot), and *C. ficifolium* Sm. (fig leaf goosefoot), were reported to harbor the pathogen based on morphological identification [39,45,57] and molecular COX2 bar coding for *C. berlandieri* var macrocalycium (Table 2). These reports require further investigation to confirm the accurate identity of the pathogen. Cross-infection reported so far is solely that of *P. variabilis* isolated from *C. album* and *C. quinoa* [52].

### 2.2. Infection Biology and Disease Symptoms

**Figure 2:**
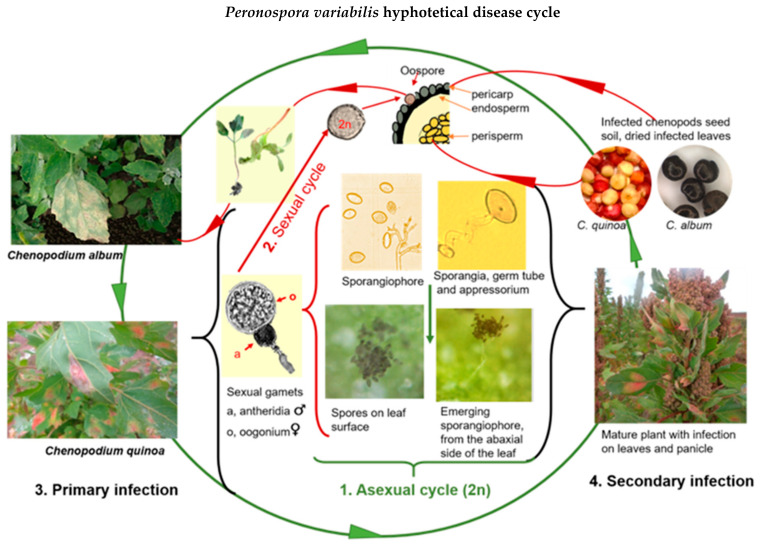
Proposed disease cycle of quinoa downy mildew caused by *Peronospora variabilis* (Photos: C. Colque-Little). Picture of haploid gamets adapted from Judelson [58].

Based on various scientific studies, we assembled a hypothetical disease cycle for *P. variabilis* (Figure 2).

**Figure 3:**
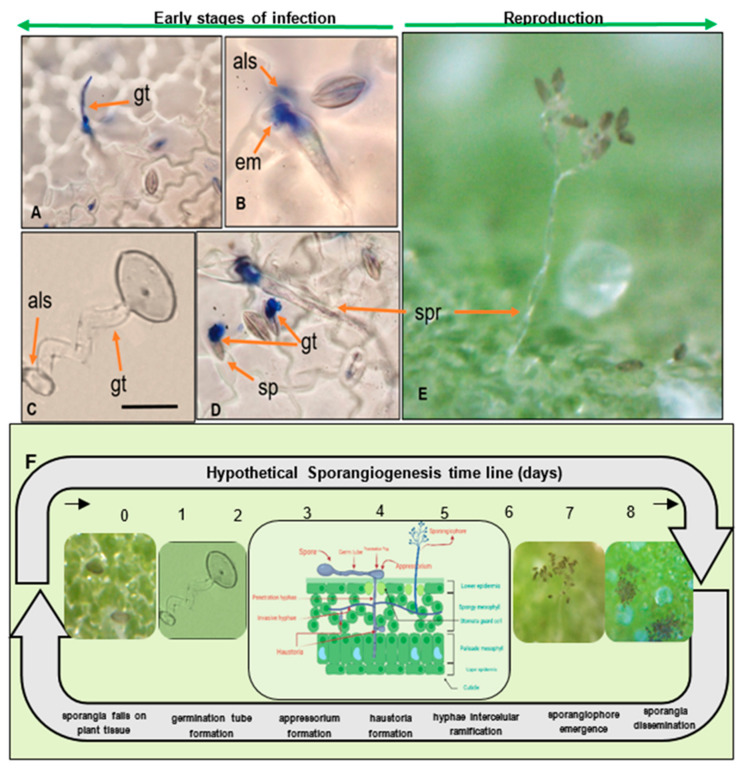
Quinoa leaf infections caused by *P. variabilis* sporangiogenesis during the early stages of asexual reproduction. (**A**) Sporangium forming germ tube (gt) and faint penetration hyphae towards the mesophyll. (**B**) Extracellular matrices (em) secreted from germinating sporangium (sp) and appressorium-like (als) structure penetrating stomata. (**C**) Sporangium, forming germ-tube (gt) and appressorium like structure in water. (**D**) Sporangiophore (spr) emerging from stomata. (**E**) Sporangiophore holding sporangia, emerging from lower epidermis. Scale bar: 20 µm. (**F**) Hypothetical *P. variabilis* sporangiogenesis timeline (Photos: C. Colque-Little). Illustration in timeline created with Biorender.com.

When mature sporangia fall on compatible leaf tissue with free moisture and relative humidity (more than 85%), the infection begins. Spores from pathogenic oomycetes produce an adhesive vesicle on the spore side in contact with the host (ventral) at early infection stages (Figure 3F). Next, a germ tube that faces the host is produced and grows chemotropically toward a suitable penetration site. In most downy mildews, the hyphae enter the leaf via stomatal pores [58] (Figure 3C). The formation of an appressorium-like swelling (penetration structures that exert pressure) on histopathological samples was observed under a microscope [52,59]. It penetrated the stomata (Figure 3B,D) but did not directly penetrate the cuticle [49,59]. Spores are chemotaxically and mechanically dependent on the stomatal aperture [60,61].

Stomata colonization happens relatively quickly. Once an appressorium is established, the secretion of extracellular matrices during the germination of the sporangia appears, as reported elsewhere (Figure 3B) [59]. The hyphae ramify intercellularly, forming haustoria (feeding structures) through the leaf tissue five to six days after penetration (Figure 3A–F). The sporangiophores emerge from the leaf’s surface around the seventh day, carrying asexual lemon-shaped sporangia (Figure 3E,F). Seven to ten days after the primary infection, sporangia are disseminated to other leaves by wind and water [40,59] (Figure 3F). They are assumed to be of importance for spreading the disease during the growing season at this stage [62]. In general, *Peronospora* species require moderate temperatures (10 °C–20 °C) for optimal sporulation [63,64]. While the disease is developing, several asexual cycles (reproduction of sporangia) may occur. Secondary infection demonstrated that the disease could spread rapidly in the field if the optimal conditions are present [54].

**Figure 4:**
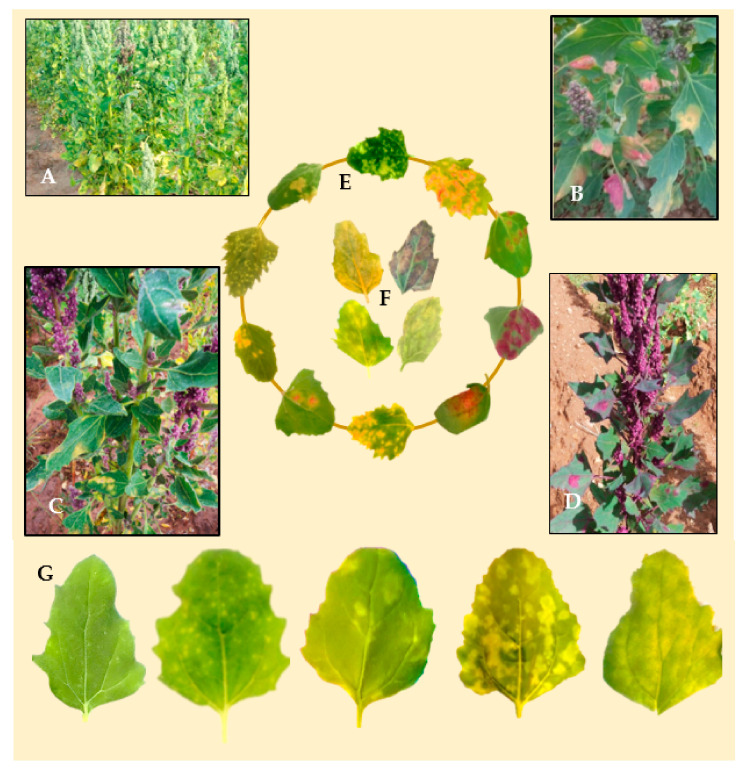
(**A**) Quinoa crop severely damaged by downy mildew. (**B**–**D**). Infected varieties in the fields of the main quinoa growing areas of Bolivia. (**E**) Adaxial leaf side belonging to different quinoa genotypes artificially infected with downy mildew. (**F**) Abaxial side of the leaves showing sporulation. (**G**) Differences in disease symptoms, ranging from hypersensitive reactions causing pale yellowish spots (**left**) to high susceptibility with chlorotic lesions covering the whole leaf (**right**) (Photos: C. Colque-Little).

Infected leaf tissue manifests lesions and signs on both sides of the leaf. Sporulation becomes apparent mostly on the leaf surface. Symptoms on infected plants vary depending on genotype, growth stage, and environmental conditions (Figure 4A–D). Classic symptoms include pale or yellow chlorotic lesions on the leaf surface (Figure 3E) and dark gray-violaceous sporulating areas, mostly on the lower surface (Figure 3F). The lesions can be several and small in some cultivars, whereas in others the lesions are extensive, diffuse, and irregular (Figure 4G). Lesions turn pink, red, purple, or light-brown, depending on the plant’s pigments (red-violet and yellow betalains [65,66]). A hypersensitive response has also been observed (Figure 4E,G). The sporulation presence differs considerably, probably due to cultivar responses and the pathogenic capability of the specific isolate [52,54,67].

Downy mildew primarily affects the foliage, but it is possible to find it colonizing different organs and tissues of quinoa plants. However, its symptoms are less obvious and sporulation is inexistent. Therefore, polymerase chain reaction (PCR) was used to amplify *P. variabilis* DNA. Taha (2019) gathered a composite of quinoa seedlings at different growth stages, subdivided them into different organs, and detected *P. variabilis* DNA on 0.8% of the root samples, 83% on the cotyledon and leaf, and 42% on steam samples. The PCR was also positive for 60–80-day-old plants’ inflorescences [68]. In addition, scanning electron microscopy was capable of visualizing *P. variabilis* on petioles [59], and the mycelium was seen as in the intercellular spaces of the leaf midrib of 80-day old plants [69]. Since the pathogen was detected at early and late growth stages of the quinoa plant, it was thought to present a systemic mode of infection [68]. However, other researchers argue [69] that the germinated oospores-mycelium spreads through intercellular parenquimatic spaces (next to xylem but not wood vessels) of the hypocotyl acropetally, towards the plant’s aerial parts, and is finally inserted into the developing seed. For clarification of the mode of infection of *P. variabilis*, more research is needed.

### 2.3. Morphology and Reproduction

**Figure 5:**
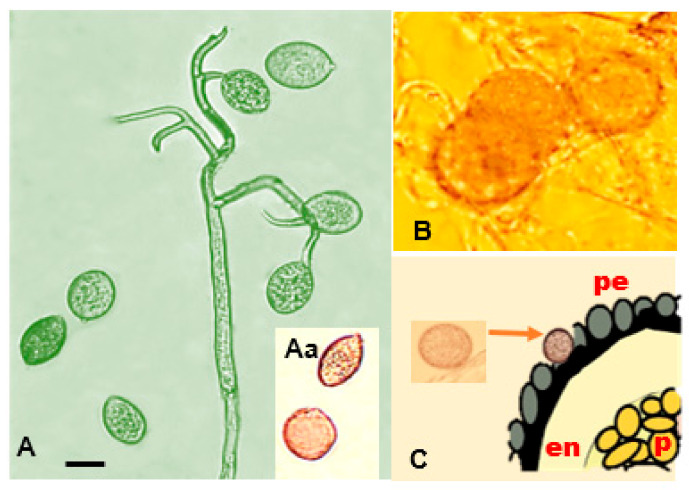
*Peronospora variabilis* spores isolated from *C. quinoa.* (**A**) Sporangiophore with lemon-shaped sporangia. (**Aa**) Oospores–sporangia size comparison. (**B**) Oospore on top of dried leaf tissue. Scale bars: 20 µm (Photos: C. Colque-Little). (**C**) Schematic representation of oospore localization in quinoa seed. o = oospore; pe = pericarp; en = endosperm; p = perisperm (illustration adapted from Danielsen and Ames (2004) [54] and Prego et al. (1998) [78]).

*Peronospora variabilis* hyphae are coenocytic (hyphae without septae) and multinucleate, resulting from nuclear divisions within the cell without an accompanying division of the cytoplasm (cytokinesis). Sporangiophores are 240–580 µm long, slender, arborescent, dichotomously ramified five to six times in a sharp angle, ending in two to three straight to slightly curved branches (Figure 5A). Ultimate branchlets are in pairs or single, flexuous to curved 8–23 (av. 12.3) µm long, with obtuse tips (Figure 5C) [29]. Sporangia are pedicellate, deciduous, olivaceous with a grayish tint, broadly ellipsoidal to ellipsoidal (av. 27.7 µm long × 21.0 µm wide (Table 3), ending in an apical translucid papilla [47]. Taxonomic measurements such as spore lengths and widths can vary depending on the homogeneity of the conidium population, the origin of the isolates, the spore subpopulation, or different roles or times in the pathogen’s life history [70]. Measuring that variability under the microscope allows researchers to estimate the mean length/width with a reasonable level of resolution when a minimum of 41–71 spores are measured for the *Peronospora* genus [71]. Even though *P. variabilis*, infecting *C. quinoa* and *C. album*, is conspecific [56], sporangia found in *C. album* were slightly bigger. Further research is needed to figure out why this difference exists. Table 3 illustrates this variability from measurements taken by various researchers [26,29,31,46,47,49,51,54,72] and the average of their measurements is provided as a reference (Figure 5B,D).

*Peronospora variabilis* can reproduce asexually (sporangiogenesis) and sexually (oospore formation and germination). It has been reported to be heterothallic and requires two compatible partners for oospore formation (mating). When eight single-lesion isolates coming from different regions of Peru and Bolivia were crossed in all possible combinations using a detached leaf assay, the existence of two mating types, P1 and P2, was apparent [73]. Sexual cycles start with a male (antheridium) and a female (oogonia) gametangia. These structures can be observed in the leaf mesophyll of plants sown 45 days earlier [69] and have the appearance of swollen hyphal tips [74]. Once in contact, both swell, especially the oogonia. Next, synchronous meiosis occurs within each one, and a pore develops between them. A single haploid nucleus is then transmitted from a male to a female. After fecundation, the development of an oospore starts by establishing a thick multi-layered wall. During maturation, the ribosomes and cytochromes disappear. The combination of their lowered metabolism, thick wall, and lipid-rich cytoplasm make them effective resting structures. Walls are usually hyaline, yet contain a brownish pigment, and their thickness ranges between 3 and 6 µm in most *Peronosporales* [58]. *Peronospora variabilis* oogonia (isolated from *C. album*) are subglobose with an average diameter of 43.5 µm [26,31]. The oospore shape is globose to ovoid; their color varies from transparent to golden brown to brown [53,75].

Ooospore diameter has been reported to range from 18.2 to 44.5 µm on average [53,69,75] when isolated from *C. quinoa*, compared to 25 to 44.5 µm on average when isolated from *C. album* [26,54]. These differences may be due to interactions with the host, environmental conditions, the age of the spore, or the pathogen races [53] (Figure 5 Aa,D). Oospores can survive inhospitable environments, such as freezing, desiccation, starvation, and microbial degradation [19]. They permit the completion of the pathogen life cycle and enhance its fitness by providing a mechanism for genetic variation [58]. Resting structures are often the source of initial infection. El-Assiuty (b) et al. (2019) hypothesize that oospores bearing tissues (cotyledons, leaves, and the perianths of seeds) shed during the life cycle of quinoa plants may play a role in the persistence of oospores in soil.

Danielsen and Ames (2004) [54] detected oospores in the pericarp (external tegument of the episperm) using ultra-microtome cuts (Figure 5F). El-Assiuty (a) et al. (2019) confirmed their occurrence in examined seed samples, revealing a 90% presence in the perianth, 87% in the seed coat, 3% in the embryo, and 2% in the perisperm [53].

To follow the passage of *P. variabilis* inside tissues, El-Assiuty (b) et al. (2019) conducted histopathological/microscopic investigations at different plant growth stages. After planting the surface-sterilized seed of a downy mildew susceptible variety, the observations started. Oospores were present in the radicle-pith three days after germination, inside the cortex of hypocotyls, and in the mesophyll of cotyledons seven days after planting. Oospore germination started with two undulating germ-tubes located opposite to one another. They develop in the cortex tissue of juvenile seedlings 15 days post-planting [69]. This research is consistent with what has been found for other downy mildew diseases such as *Plasmopara viticola*, the mature oospores of which germinated for 3–7 days under a favorable regime of rainfall and temperature [76].

Moreover, oospores were detected in all tissues of quinoa plants that had been sown 45–120 days previously [53,69]. They were also seen on leaves of senescent infected plants, artificially inoculated with a single Danish isolate under greenhouse conditions, suggesting that the isolate had both mating types present (Figure 5E. Colque-Little, unpublished). In addition, they have been found in old infected leaves collected in Andean regions of quinoa production (Peru, Bolivia) [54] and fresh leaf tissue collected in Pennsylvania, USA [77].

Greenhouse experiments with oospore-infected seed samples sown in high and low relative humidity showed a significant difference in visible seedling infections among samples under high humidity and with a large oospore density in most cases. However, oospore density seems to be more critical for seedling infections when the relative humidity is low [75,78,79]. The number of oospores can be estimated using the seed washing method [54,80,81]. Briefly, the seed is soaked in water under agitation. Seeds are removed with cheesecloth, the solution centrifuged, the supernatant discarded, and the pellet is dissolved in sterile water. The number of oospores is counted using a hemocytometer under the microscope. Calixtro (2017) quantified the number of oospores present on susceptible seeds and found it was three times greater than the number on tolerant varieties demonstrating that host genotype is an important factor [82].

### 2.4. Peronospora variabilis Genotypic Diversity and Virulence Profiling

*Peronospora variabilis* is a genetically diverse group [30] with multiple population structures, in light of three facts:

1. Chenopod hosts have a vast degree of genetic diversity and plasticity [83,84].
2. *Peronospora variabilis* has great adaptability (climatically and geographically), hence its worldwide geographic presence [54].
3. The occurrence of sexual reproduction permits genotypic pathotype expansion [4].

Quinoa cultivation areas of the Andean region have resulted in severe infections under field conditions. Swenson (2006) collected 43 isolates from eight Bolivian regions [44]. Phylogenetic fingerprinting relationships revealed high genotypic diversity within a geographical region. The most recent fungal and oomycete identification initiatives were carried out using DNA sequencing [12]. A group of *P. variabilis* herbarium and isolates from different geographic locations (Argentina, Bolivia, Denmark, Ecuador, and Peru) were phylogenetically analyzed based on ITS rDNA sequences. The majority of the Danish and South American isolates were separated into two major clusters [29]. *P.variabilis* was detected in 31 out of 33 quinoa seed lots destined for human consumption and originated in six different countries. Subsequently, ITS and Cox2 phylogenetic relationships were examined to determine whether geographical differences occurred. ITS-derived phylogeny showed no genetic differences, but the Cox2 phylogeny indicated that geographical differences existed between US and South American samples [32]. In another study, researchers characterized 40 isolates from *P. variabilis* originating in the Andean highlands (Peru and Ecuador) and Denmark (Jutland, Sealand) using universally primed PCR (UP-PCR) fingerprinting analysis. A separation between the Danish and Andean isolates in two distinctive clusters was found, together with genotypic variations between isolates within each cluster [30].

In the future, the next step might be the virulence profiling of *P. variabilis*, achieved through the sequencing of its genome, followed by transcriptomic analysis. Progress in genome sequencing technologies can provide genome data to better understand how microbes live, evolve, and adapt. Indeed, the genome of three races of *P. effusa* (downy mildew of spinach) was recently sequenced, assembled, and annotated to gain insights into its gene repertoire and identify infection-related genes [9]. The genomes of microbial pathogens can vary greatly in size and composition; this also includes when closely related species are considered. In the case of *Peronospora*, species greatly vary between 45.6 to 159.9 Mb when estimates are made using image analysis of nuclear Feulgen staining [85]. Whether genome sizes have an impact on the lifestyle of *Peronospora* species is still unknown [86].

Another way to elucidate genotypic and phenotypic variation within pathogen populations is to use virulence-phenotypic assays with a standard set of differential hosts. Spinach downy mildew has such a set composed of 11 cultivars, maintained with the help of the international working group on *P. effusa* (IWGP) [86,87,88].

This organization invites researchers to use the set to identify new isolates that can later be nominated, tested for various criteria, and then given a race designation [86,87,88]. An international system for monitoring the virulence of *P. variabilis* has not yet been developed. However, Ochoa et al. (1999) made the first step towards this from a collection of twenty *P. variabilis* isolates that corresponded to different Ecuatorian ecoregions [40]:

1. An area where quinoa cultivation was not regularly practised. The least virulent strains were present here and were identified as virulence group 2 (V2).
2. A region where landraces and newly released cultivars were introduced. Only the most virulent strains belonging to group 4 (V4) were present here.
3. Fields located where landraces and newly released cultivars have been cultivated for many years. Here, all four virulence groups were present.

**Figure 6:**
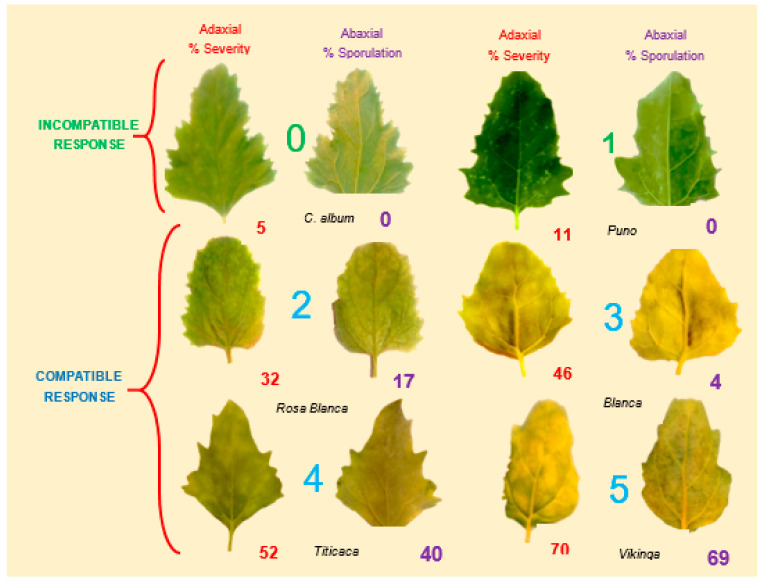
Set of reference cultivars postulated for profiling the virulence of *Peronospora variabilis,* including *C. album* and two Bolivian cultivars with intermediate reactions. Leaves from three-week-old artificially inoculated plants. Numbers in red indicate the percentage of severity on the adaxial side, and those in purple indicate the percentage of sporulation on the abaxial side [52]. Numbers in green (incompatible response) and blue (compatible response) correspond to Ochoa’s scale: 0 = no symptoms; 1 = 2−5-mm lesion with truncated mycelium in the mesophyll of the leaf; 2 = 4−8-mm chlorotic lesions with minor sporulation; 3 = medium-sized and confined chlorotic lesions with sporulation mainly on the abaxial side of the leaf; 4 = large, not clearly confined chlorotic lesions with sporulation mainly on the abaxial side of the leaf; 5 = mild chlorosis with abundant sporulation on both adaxial and abaxial sides of the leaf (Ochoa et al., 1999 [40], Colque-Little et al., 2021 [52]). Both assessments are comparable in terms of severity and sporulation; thus, the existence of resistance factors is hypothesized in this set of reference cultivars.

Ochoa et al. (1999) investigated seedlings under controlled environments from 60 selected genotypes and the above-mentioned *P. variabilis* collection; quinoa lines were selected for consistent compatible/incompatible reactions. Based on these results, four resistance factors (R1, R2, R3, and R4) were postulated [40]. It was most likely that two mating types are present. areasall difference exists. However, these genotypes are exclusive to the National Ecuadorian collection and thus not available for research. The measurements of severity and sporulation of downy mildew from reference cultivars (Puno, Titicaca, and Vikinga) and many other genotypes used by Colque-Little et al. (2021) are comparable to the 1–5 scale developed by Ochoa et al. (1999) (Table 4 and Figure 6). Therefore, we suggest that the presence of resistance factors could be preliminarily hypothesized on reference cultivars. Importantly, the seed of these cultivars is commercially available (Quinoaquality.com, Denmark) and could be established as an international reference set.

### 2.5. Disease Assessment under Controlled Conditions and in the Field

Reliable identification, followed by the assessment of disease, is the first step in efficient management. It is also an important component in the development of disease-tolerant quinoa varieties. It allows for crop-loss assessments and screening for host–pathogen interactions. Assessment methods must be in close agreement with the goals of the trial(s). Evaluations might differ according to the experimental setup. For seedlings, detached leaves and plantlets under controlled conditions and a disease assessment scale can be used (Figure 6). For the assessment of diseased plants in the field, it is necessary to take into account:

1. The phenological stage of the plants. Age-related resistance becomes relevant for biotrophic pathogens, which require healthy plant tissue to complete their cycle. [89,90]. The observation of symptoms should reflect the progression of the disease through periodical records, rather than observing its percentage of occurrence or incidence. For the quinoa/downy mildew interaction, it has been demonstrated that disease incidence has a low heritability *H*^2^ = 0.4 and a low correlation with severity and sporulation (0.67 and 0.65, respectively) [52]. Therefore, incidence or whole plant scores are unsuitable for this type of trial. To measure the area under the disease curve progression (AUDPC), a minimum record of three to four observations of disease severity is essential. A similar study has highlighted the importance of measuring the disease severity over time for other interactions, such as *Phytophthora infestans* infecting potatoes. The objective is to capture low, medium, and high infection levels in all the genotypes, including the susceptible ones [91].

Calixtro (2017) recorded high variability in the area under the disease progress curve (AUDPC) within the same quinoa accession during different phenological stages. The higher AUDPC values were seen at 104 days after sowing with favorable disease conditions [82].

Therefore, we suggest assessing downy mildew as soon as the first symptoms of the disease are visible. The first reading could be when nine pairs of leaves (BBCH 1–1.9) have emerged or beforehand in cases where disease symptoms are visually observed. Time intervals among subsequent readings depend on whether the disease advances slowly or quickly [91]. Other observation points could be during development (BBCH = 4) or visible inflorescence (BBCH = 5–5.9) and the last one at complete anthesis (BBCH 6–6.9). The phenological growth stages mentioned here correspond to the international quinoa-based coding system BBCH (Biologische Bundesanstalt Bundessortenamt und Chemische Industrie) [92].

**Figure 7:**
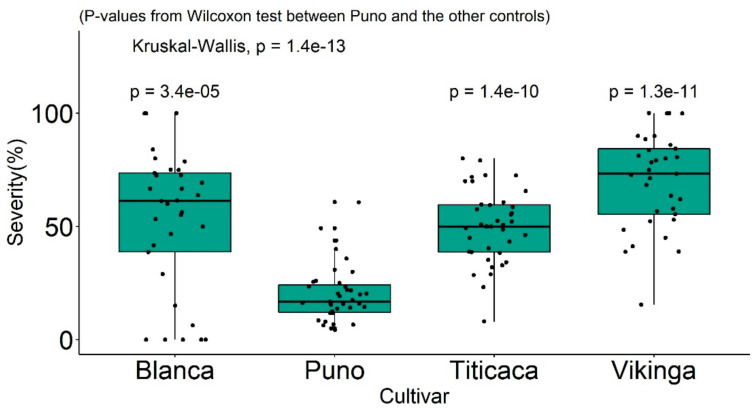
Reference cultivars’ responses to infection with *Peronospora variabilis,* measured in mean severity under greenhouse conditions. Source: Colque-Little et al. [52].

2. The vegetative cycle of the plants. Late-maturing quinoa genotypes will display some degree of resistance [93] by increasing the latent period of the pathogen. Thus, readings for severity were taken ten days after infection instead of five in a recent study, in which a late cultivar Blanca was compared with the Danish cultivars Puno, Titicaca, and Vikinga [52] (Figure 7). Puno matures ten days later than Titicaca [94]. Cultivar Blanca is considered susceptible when additional time is given [52] (Figure 7). Vegetative cycle effects were also shown in another study that analyzed the mean-based cluster of inter-ecotype F2:6 population crosses and identified the following three clusters [48,95,96]:
  (a) Cluster one: consisting of late, mildew-resistant, high-yielding lines;
  (b) Cluster two: consisting of semi-late lines with intermediate yield and mildew susceptibility;
  (c) Cluster three: consisting of early to semi-late accessions with low yield and mildew susceptibility.

Therefore, for a proper comparison, quinoa lines with similar vegetative cycles should be screened in the same experiment or statistical adjustments should be carried out as part of the analysis. In addition, a positive control (susceptible variety) and a negative control (resistant variety) might be beneficial in the analysis.

**Figure 8:**
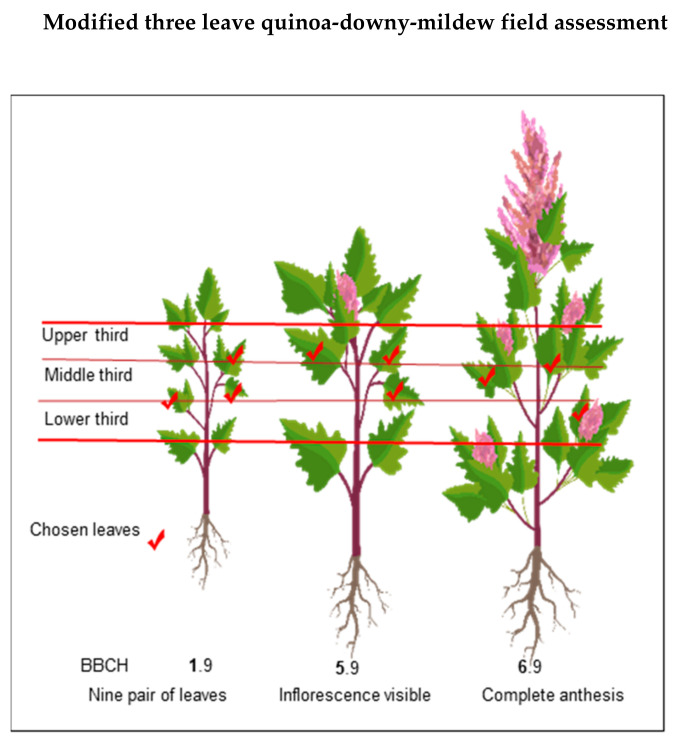
Modified from Danielsen and Munk (2004) [97]. Three-leaf field assessment method for quinoa-downy mildew at different growth stages.

**Figure 9:**
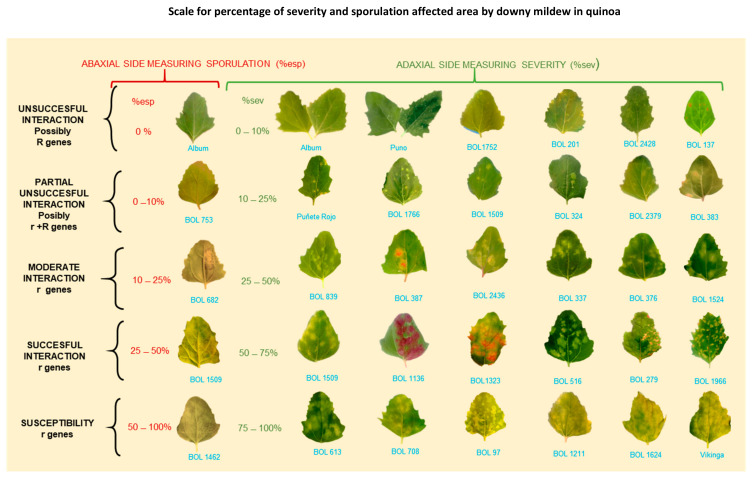
Scale for percentage of severity and sporulation area affected by downy mildew in quinoa. r = postulated minor genes; R = hypothesized major genes. BOL = accession numbers. **Note**: Percentage of sporulation is estimated on the abaxial leaf side area covered by visible lesion. It is not estimated on the total abaxial side leaf area (Colque-Little et al., 2021) [52]. Photos by Colque-Little.

3. Sampling method and sample size. Depending on the size of the experiment, there is no need to take severity readings in all the quinoa plants. Instead, consider the plot level and take readings on representative samples. Normally, 6–10 plants per plot are sufficient [54,91]. Next, an estimation of the percentage of affected foliage is required. Given the size of the plants and abundant foliage, it is not feasible to analyze the entire foliage; thus, it is recommended to perform scoring on individual leaves of the chosen plants [54]. Danielsen and Munk [97] evaluated various field assessment methods to predict yield losses due to downy mildew. The three-leaf method resulted in the highest negative correlation to yield (r = −0.736). Furthermore, disease progression relies on the successful infection of the host. It is often assumed that the susceptibility of host tissue is constant. However, in reality, it is a function of plant age and leaf position [14,98,99]. These responses might result from inducible plant defense responses, which occurs at the starting interaction site but also in distal, uninfected parts [99,100,101]. For these reasons, we suggest randomly choosing three leaves from the middle part (lower third, middle third, and upper third), as illustrated in Figure 8. Avoid the lower and upper extremities of the plant because they are prone to senescence/defoliation [97] and plant defense responses, respectively. Next, estimate the percentage of affected leaf area using the attached scale [79] (Figure 9). The average value from the score of the three leaves becomes the percentage of severity for each plant.

### 2.6. Yield Losses and Management

The losses caused by downy mildew depend on the plant’s phenological phase at the time of infection and the amount of resistance that the cultivar has [102]. Infection of susceptible cultivars may result in severe yields losses if the pathogen has favorable weather conditions, particularly high relative humidity [54]. If the infection occurs in the plant’s initial growth stages, susceptible crops could completely fail; in less susceptible cultivars, the loss may fluctuate between 20% and 40% [4]. In a conventional intensive agriculture system of Cajamarca (Peru), between five to seven fungicide applications were needed to control the infection during the agricultural campaign [103].

Due to the high capability of *P. variabilis* for the proliferation and latent infection on *C. quinoa* and *C. album*, the scenario for low-input farming has only two options for disease control:

1. tolerant crop varieties; and
2. cultural practices (options on the list below).

Alternative cultural practices:

(a) Policymakers, smallholder farmers, and other stakeholders need resources for collective action for the establishment of a seed supply chain with quality standards (low levels of key seed-borne diseases). Experiences with complementary intervention such as capacity building and technical assistance have shown this influence in an appropriate conceptual model of sustainable production [104].
(b) The detection of *P. variabilis* on the seed is achieved using a simple method [32,54]. In the case of the presence of an oospore, treat the seed with a systemic fungicide [105]. For small samples, alternative treatments such as a hot water bath (50 °C–60 °C) could be considered for 10 to 30 min, as this method has been applied successfully to eradicate seed-borne pathogens of spinach [106]. After or without treatment, the addition of beneficial microbes by priming the seed with products such as commercially available *Trichoderma* can enhance the growth of the plants [107].
(c) Adjusting the space between rows and individuals, making the area less dense and increasing space between plants. In areas where the RH is as high as 80%, the minimum should be a 0.5-m space between rows and 0.15 m between plants [5].
(d) Avoiding excess water in the field;
(e) Implementing effective weed control, especially of alternate host *C. album*;
(f) Practicing crop rotation;
(g) Spraying the plants around 45 days after planting in areas with endemic infection as a preventive measure [69]. Use oomycete sensitive chemical control measures (e.g., Alietti) at principal growth stages, e.g., leaf development, inflorescence emergence, flowering, and fruit development [14,54,92]. Fungicides could be applied, alternating between systemic and contact products, starting with systemic products. Bio-pesticide or plant extracts could replace fungicides with a uniform and preventive application [5]. Inducers of resistance are an alternative [108].

### 2.7. Genetics of Resistance to Downy Mildew

**Figure 10:**
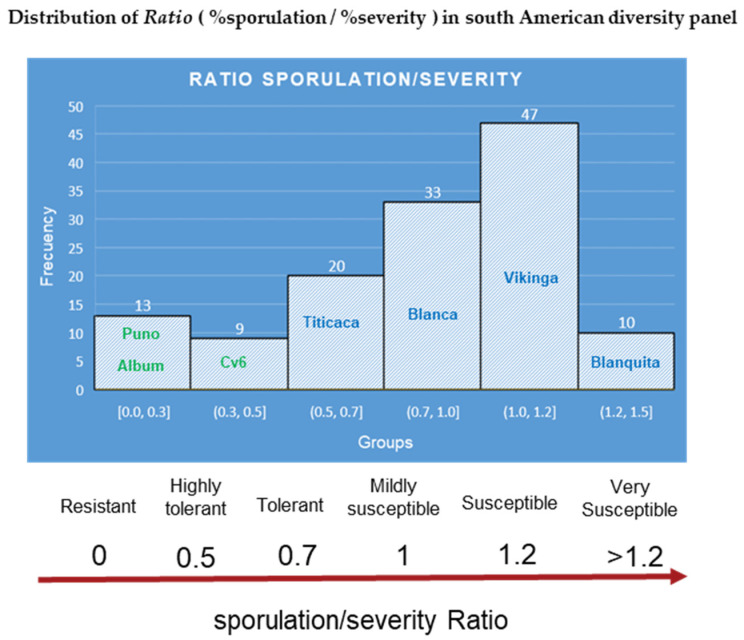
Ratio calculated from mean averages of sporulation/severity for the South American diversity panel. The names inside the histogram bars correspond to reference and representative cultivars for each group. Source: calculated with the data set from Colque-Little et al. (2021) [52].

For agriculture, field or host resistance is still the most important way of controlling diseases because it leads to the most cost-effective ratio for the grower [109,110,111].The response to downy mildew in a diversity panel of 132 quinoa genotypes resulted in strong phenotypic variation with high disease trait heritability (*H*^2^ = 0.78 for severity, *H*^2^ = 0.82 for sporulation). This variability was paired with the analysis of 603,871 SNPs in 61 genotypes with FarmCPU [52]. A single variant on chromosome 4, located above a threshold with a lack of siginificant marker trait wide associations. A single variant on chromosome 4, located above a threshold with a lack of significant marker-trait wide associations, suggested a polygenic architecture for the downy mildew interaction in agreement with other studies [43,48,49,95,104,112,113,114,115]. However the interactions of the host resistance pathway with a biotrophic pathogen (e.g., *P. variabilis*) are complex. The interaction oscillates between compatible (susceptible) and incompatible (resistant) states, because the genes involved can introduce quantitative variations, adding different levels of reactio to the extreme responses adding different levels of reaction to the extreme responses [116]. The same study phenotyped hypersensitive responses, most probably corresponding to R-genes, and very low sporulation on resistant genotypes, which could correspond to defeated R-genes [52] (Figure 3E,G). Indeed, Gabriel et al. (2012) characterized the quinoa/downy mildew pathosystem in field experiments and discussed the presence of R-genes, multiple r genes, defeated R-genes, and combinations, with the most common interaction being that corresponding to field resistance [93]. The deployment of different genes depends on many factors, such as pathogen isolate aggressivity [40] and environmental conditions. For quinoa downy mildew, it was demonstrated that the variance for genotype-by-experiment interaction σ2 G E was large, reflecting that even minute environmental changes can trigger a genotype to respond differently to the disease (Figure 10) [52]. The degree of resistance that the plant displays is determined by these changes interacting with the host genetic composition [116]. Furthermore, segregation in an F2 mapping population derived from a cross of saponin-free and bitter genotypes suggested that downy mildew resistance has a dominant inheritance [117].

Therefore, field phenotyping experiments of *P. variabilis* infections using diverse quinoa genotypes should include multiple environments and points in time. Using mixed modeling to detect quantitative trait loci (QTLs) by considering them as random samples from a population of target environments and time could be one alternative [118]. Under controlled conditions, it would make sense to use elite diversity panels with replicates, reference cultivars, and genetically diverse pathogen isolates in a series of experiments that are designed randomly.

**Figure 11:**
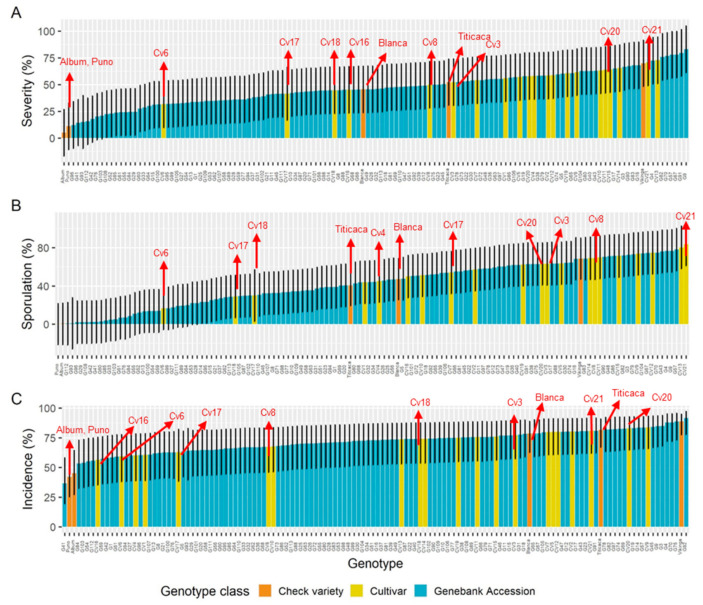
Disease traits estimated means fitted on a generalized linear mixed model (GLMM) for a diversity panel, comprising gene bank accessions (landraces), cultivars (Bolivian-bred cultivars), and check varieties (reference cultivars). (**A**) Severity of infection, (**B**) sporulation, and (**C**) incidence of infection. Error bars represent 95% confidence intervals. Adapted from Colque-Little et al. (2021) [52].

The characterization of a south American panel demonstrated robust differences between the genotypes for all disease traits [52] (Figure 11). Moreover, at least five cultivars that were released by Bolivian breeding programs [112,119], which included downy mildew tolerance, showed moderate to low severity and reduced the reproduction of the pathogen. Interestingly, the incidence (Figure 11A,C) and severity of cultivars 6, 17, and 18 might have classified them as susceptible, but their ability to prevent the pathogen from multiplying conferred them some degree of resistance [110] (Table 5). Indeed, the Danish variety Titicaca was classified as susceptible through the solely detached leaf sporulation assay [48]. When the assessment was done as a function of both parameters, by calculating the ratio (*R* = %sporulation/%severity), Titicaca’s *R* = (40/52) = 0.77 showed that it is not completely susceptible (Table 5). This finding suggests that the scoring of both parameters in plantlets can contribute to better disease assessments of cultivars.

Therefore, we propose using the (R = % sporulation/% severity) ratio to better rate elite genotypes in breeding programs. Using the data set from a previous study [52], the ratio was calculated. Histograms separated the diversity panel into six groups and derived a ratio-based scale (Figure 10). The bimodal distribution displayed by the histograms is consistent with previous findings for *P. variabilis* field interactions [44].

Quinoa cultivation in South America occurs in agro-climatological polar regions. These regions have been classified according to their soil type, rainfall, and temperature as Northern, Central, Southern highlands, and Andean slopes (Table 6) [4,96,112,119]. The Andes have heterogenic topography; their altitude ranges between 3200 and 6500 m above sea level; hence, there are variations in temperature and humidity [120]. Indeed, temperature decreases at a rate of 0.7 °C for every 100-m increase in altitude in Chile’s Tarapaca region. Therefore the coastal Atacama littoral plains differ from mountain sites (e.g., Los Condores) which enjoy fog oases and lomas vegetation [121]. A similar situation is expected for the slopes in the Andes of Bolivia, Peru, and Ecuador.

**Figure 12:**
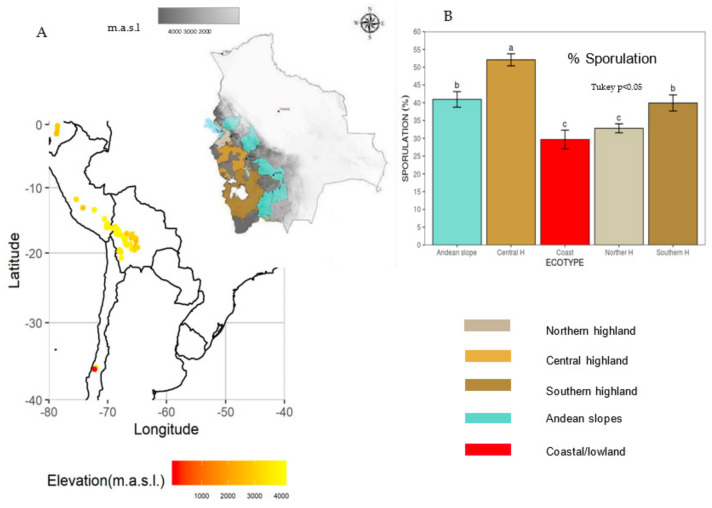
(**A**) Modified map of germplasm bank accession across South America by elevation Source: Colque-Little et al. (2021) [52] and modified map of Bolivian ecoregions for quinoa production. Source: Gandarillas et al. (2015) [6]. (**B**) Mean sporulation on diversity panel related to quinoa ecoregions calculated with Tukey test (*p* = 0.05). Different letters (a,b,c) represent significant differences between the sporulation produced by genotypes coming from different ecoregions when infected with *P. variabilis*.

Quinoa ecoregions were inferred from information provided by passport data (germplasma.iniaf.gob.bo-GRIN global, accessed 15 April, 2020) and the characterization of Bolivian and Coastal ecoregions [112,120,121]. The information is summarized in Table 5. Disease traits data (mean values of severity and sporulation) from the South American diversity panel [52] were analyzed with the Tuckey test for their relationship with the seed-ecoregion collection site. For this analysis, we used Inti-Yupana for R [122], and the results pointed at significant differences for the variables. The graph represents data from means of sporulation only because data from means of severity was very similar (Figure 12).

Even though the sample size from the central highlands was overrepresented and the Coastal sample size was underrepresented, significant differences (*p* = 0.05) for severity and sporulation were detected. The most resistant genotypes from the South American diversity panel came from the coastal/lowland and northern highlands ecoregions. The northern highlands are the most humid since they are close to Lake Titicaca. This ecoregion is suitable for pathogen infections and disease pressure. This outcome is in agreement with previous reports [4,48,54,93,95]. The Danish cultivar Puno was found to be resistant, as reported elsewhere [48]. Moreover, principal component analysis of genome-wide association studies (GWASs) demonstrated that Puno is genetically close to Chilean coastal lines and separated from highlander genotypes [52]. The central highlands showed the largest quantity of susceptible genotypes, likely because they were also the most numerous. However, a few genotypes with a large amount of sporulation came from the southern highlands of Bolivia (i.e., G16, G17, and G82) [52] (Figure 11B and Figure 12B).

In conclusion, the genetic improvement of quinoa for downy mildew tolerance is possible because resistance is present in multiple genotypes, but a virulent pathotype might overcome it. Other options to consider are discovering, transforming, and deploying resistant alleles existent in wild species such as *C. albums* [52,123,124]. Because tolerant varieties seem to delay and reduce the disease progression, inducers of resistance [125,126] could be a feasible option [108].

## 3. Ascomycete Fungi

### 3.1. Fungi Identified by Molecular and Morphological Approaches

#### 3.1.1. Ascochyta Leaf Spot and Black Stem (*Ascochyta hyalospora* and *A. chenopodii*)

At least two *Ascochyta* species infect quinoa, causing quinoa leaf spot (described below) and black stem (described in next section). Quinoa leaf spot is either caused by *Ascochyta hyalospora* or *A. chenopodii*. *A. hyalospora* Coole and Ellis is an *Ascomycete*, class Dothideomycetes, order Pleosporales. It was first found as a seed-borne pathogen of *C. quinoa* from the Bolivian central highlands, for which a blotter test (seed incubation method on well-soaked filter papers [127]) revealed 8%−26% of infection. It was identified morphologically, followed by pathogenicity tests causing whitish leaf spots 5 dpi, followed by pycnidia at 10 dpi, and necrosis on leaves and stem of *C. quinoa* and *C. album* plants [128]. Testen et al. (2013) isolated a fungal pathogen from quinoa fields in Pennsylvania, USA, and through DNA sequencing of the ITS1-2 region matched it to *Ascochyta sp.*, and reported that it resembled the morphological characteristics of *A. chenopodii* and *A. caulina*, which at the time of identification had no DNA bar-codes available for comparison. However, the ITS1-2 sequences from Testen et al. (2013) were not released as GenBank sequence data [129]. Thus, it is still not possible to make the comparison.

**Figure 13:**
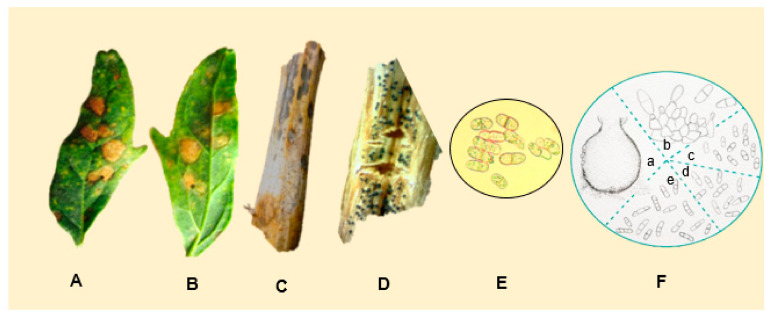
Leaves showing symptoms of infection caused by *A. hyalospora* (**A**) on the adaxial side of the leaf and (**B**) on the abaxial side. (**C**) Stems showing pycnidia and brown stalk. (**D**) Stem showing pycnidia. (**E**) *A. hyalospora* conidia (Photos: Testen, 2020) [77]. (**F**) *A. hyalospora*: (**a**) pycnidium (×200); (**b**) conidiogenous cells of pycnidium (×1000); (**c**) conidia from pycnidium (×400); (**d**) bi and tri-septate conidia from pycnidium on an inoculated stem of *C. quinoa* (×400); (**e**) conidia from pycnidium on leafspot of inoculated leaf of *C.quinoa* (×400). Source: photos (**A**−**E**) provided by A.L. Testen. F. Adapted from [128].

*Ascochyta hyalospora* pycnidia are globose to subglobose, usually 17.5 to 25 μm in diameter [128], and contain sub-hyaline to light-brown-colored conidia. The conidia are cylindrical to ovoid, measuring 19 × 7.5 μm [129] and 25 × 10 μm [128] on average. They often have one to two septa and less commonly have three septa. Boerema (1977) noted that the conidia formed on leaf spots after artificial inoculation were longer (35 μm) and often had two or three septa (Figure 13E,F). Lesions on the leaves are of irregular shape, and are bronze to reddish-brown with darker edges. Spots eventually turn necrotic. Thereafter, numerous black pycnidia, distributed randomly in each lesion, can be seen [129].

The stems show necrosis, and the pycnidia are visible to the naked eye (Figure 13A−D). The seeds turn brown, and pycnidia are observed at the stereomicroscope [128,130]. *Ascochyta* leaf spots have been considered of minor importance in the Andean region [3,4,6,131]. In 2014, large-scale cultivation (12,000 ha) of quinoa started in China [132], where the production was affected. Infected foliage decays and falls, leaving the plant defoliated [5]. Effects on quinoa production in the USA have not yet been assessed [129]. Experiments in Bolivia showed that the germination rates of seeds from infected plants were reduced by 6% to 10%. Moreover, the disease was transmitted to seedlings [5]. One possibility for control would be the use of high-quality seeds, since the pathogen is seed-borne [130].

#### 3.1.2. Quinoa Black Stem (*Ascochyta caulina*)

Molecular and phylogenetic analysis of representative isolates from quinoa black-stem revealed that its causal agent is *Ascochyta caulina* (van der Aa and van Kesteren 1979). Its sexual teleomorph stage is called *Neocamarosporium calvescens* (de Gruyter et al. 2009), previously known as *Pleospora calvescens*. The taxonomic status of *P. calvescens* has changed recently, based on multigene analyses. It has been established in the genus *Neocamarosporium* Crous and Wingfield in 2014, which comprises 15 species, including *N. betae*, *N. chenopodii*, and *N. calvescens.* These species share the same large phylogenetic branch with *N. calvescens* [133,134,135,136,137]. *Ascohyta caulina* in its asexual form belongs to the family Didymellaceae and has often been confused with *A. hyalospora* [137]. Previously, it has also been found to infect eight species of *Atriplex* and eight species of *Chenopodium*, including *C. album* [138].

**Figure 14:**
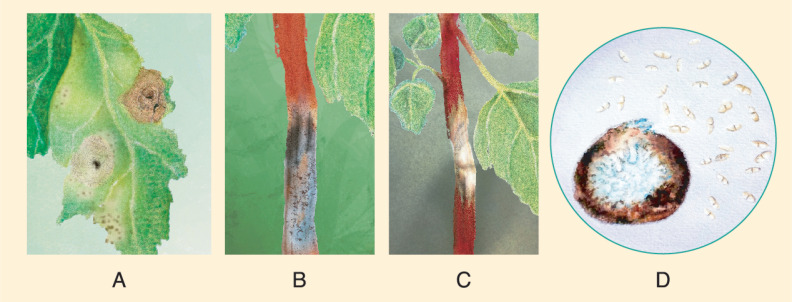
Typical symptoms of quinoa black stem in the fields of China. (**A**) Symptoms induced by inoculation of *A. caulina* on *C. quinoa* (left of midrib) and on *C. album* (right of midrib). (**B**) 10 dpi diamond shaped lesion on quinoa stem with presence of pycnidia. (**C**) Necrotic quinoa stem prior to lodging; (**D**) morphological characteristics of conidia and pycnidia of *A. caulina*. Source: illustrations based on pictures from Yin et al. [114].

Another report [139] on *A. caulina* was accomplished through a morphological description of the isolate found on quinoa seeds of cv. Cochabamba of Bolivian origin (stored at the Gene Bank of the Research Institute of Crop Production in Prague-Ruzyně). For pathogenicity tests, the isolate was inoculated in seedlings, and symptoms were reproduced. Interestingly, quinoa seeds from the University of Copenhagen, Denmark, analyzed simultaneously, were free of *A. caulina* [139]. This finding might indicate that the disease is not present in Denmark. *A. hyalospora* pycnidia are rigid structures, grayish-white or light brown, spherical or pear-shaped, and have a single chamber. They are 162 × 134 μm in size, on average. Conidia are elliptical or fusiform, light brown, oblong at the top and flat at the base, and measure 17 × 6 μm on average [137]. Conidia usually have one septum, which is erect or curved (Figure 14D). The optimal conditions for its germination are between 15–25 °C, RH = 60%. Compared to *A. hyalospora* leaf spots, black stem lesions were more likely to develop under cooler conditions [140]. Pathogenicity tests on detached stems of *C. quinoa* showed typical symptoms 10 dpi and were densely covered with pycnidia. At 15 dpi, typical symptoms appeared on the stems of plants inoculated in outdoor conditions. Detached inoculated leaves of *C. quinoa* developed visible symptoms 8 dpi and were grayish white. However, necrotic lesions are rarely seen on the leaves in the field [137] (Figure 14A).

Quinoa black stem primarily infects the stem; lesions are recorded at the flowering stage up to maturity. Symptoms first appear at the lower and middle parts of the stalk, subsequently moving upwards. They are diamond-shaped, pale or tan, and present slight depressions, as the plants are prone to drying and consequent shrinkage. The diameter of the lesion averages 7.9 cm.

The stem lesions turn necrotic in later stages and are accompanied by abundant small round protrusions of black pycnidia (Figure 14B,C). In severe cases, lesions wrap around the stem, causing lodging, foliar chlorosis, leaf abscission, and the development of “empty” and sterile grains on the panicle [137].

Quinoa black stem is considered a newly emerging disease in Chinese regions (Jingle County, Shanxi province), where the disease was severe. The incidence was around 80% and the yield was reduced by 45% [137,140]. The fungicides mancozeb and azoxystrobin are shown to have a strong inhibitory effect on conidia germination, whereas tebuconazole and difenoconazole were most effective towards mycelial growth in tests performed in vitro [137].

Sixteen European countries concentrated integrative approaches for the biological control of the weed *C. album* from 1994 to 1999. The European Research Programme (COST-816) concerted the use of a combination of *A. caulina* with ascaulitoxin for this purpose [141,142]. Experiments using *A. caulina* as a microbial herbicide were up to 70% successful in reducing field conditions, as it was able to kill its host in one week [143,144,145,146].

#### 3.1.3. Cockerel Eye/Quinoa Cercorporoid Leaf Spot

Quinoa leaf spot was first reported in Ecuador (2009) and given the Spanish common name “ojo de gallo”, or cockerel eye, because of the symptoms exhibiting a dark center and round shape. It was then associated with *Cercospora* spp. [147]. The genus *Cercospora* was established by Fresenius (1863) and belongs to the family Mycosphaerellaceae, class Ascomycota. A comprehensive list of cercorporoids assembled in Poland included a species under the name *Cercospora chenopodii* Fresenus, 1863, found on *C. album* [148].

**Figure 15:**
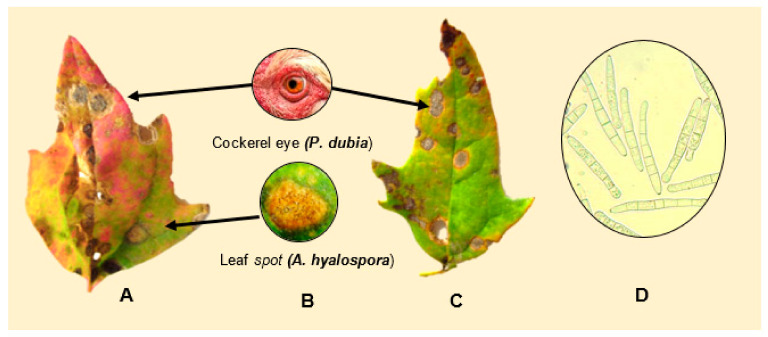
(**A**,**C**) depict symptoms of *P. dubia* on leaf tissue. (**B**) Comparison of “cockerel eye” and leaf spot symptoms. (**D**) Conidia of *P. dubia.* Source: pictures (**A**–**C**) provided by Testen and (**D**) Testen [77].

Testen et al. (2013) amplified the ITS1-2 region of strains isolated from quinoa field plots located in Pennsylvania, USA, and identified them as *Passalora dubia* (Riess) U. Braun (GenBank EF535655). Conidia were septate, hyaline, and measured 25−98 μm long × 5−10 μm wide—with an average of six cells per conidium (Figure 15D). Disease symptoms of leaves were round to oval with a diameter of less than 1 cm, and were brown to gray-black with darker brown or reddish borders (Figure 15A−C). In addition to quinoa, *P. dubia* has also been isolated from *C. album* [77,149].

A pathogen identified as *P. dubia* has been tested as a microbial herbicide for the biocontrol of *C. album* in Europe. It was shown to reduce *C. album*’s dry weight by 20% [143].

#### 3.1.4. Cercospora Leaf Spot Caused by *Cercospora* cf. *chenopodii*

**Figure 16:**
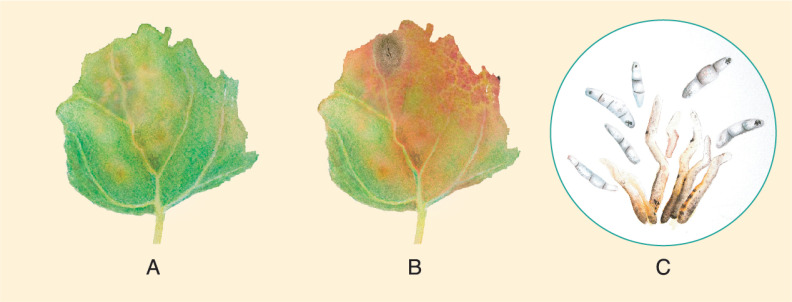
(**A**,**B**) Foliar symptoms of *Cercospora* leaf spot. (**C**) Condidia of *Cercospora*. Source: illustrations adapted from Yin et al. (2019) [151].

Cercospora leaf spot, infecting quinoa in Shanxi, China, was classified as *Cercospora* cf. *chenopodii* based on multi-loci sequencing and phylogenetic analysis using LSU rpb2 and ITS as target genes. The qualifier “cf” indicates a provisional identification [150], even though most diagnostic characteristics correspond to *C. chenopodii*. At the early onset, the lesions were nearly round and pale yellow to light brown. Later, the lesion became grayish brown, with a slightly elevated surface, a yellow halo, and an average diameter of 5.4 mm. The pathogen’s conidia were observed to be septate and hyaline to brown. They were 40.01 × 7.99 μm on average. They contain an average of four cells per conidium (Figure 16C). Spore suspensions made in glycerin causes disease symptoms 5 dpi, spreads quickly, and produces large yellow lesions, which causes defoliation 10 dpi. Optimum temperatures for infections are 22–26 °C, with a high relative humidity (75–80%) [151]. Based on multigene phylogeny (LSU, rpb2, ITS, cmdA, and other genes), various *Passalora* species have been proposed to be re-classified as *Cercospora* Fresen. *P. dubia* is included in this phylo-group and is considered synonymous with *Cercospora* cf. *chenopodii* [151,152,153].

#### 3.1.5. Quinoa Anthracnose Caused by *Colletotrichum nigrum* and *C. truncatum*

Stem lesions have been observed on quinoa plants growing in Ames, Iowa (USA). Symptoms are recognized as oval to linear, slightly narrow at the ends, light in color, silvery-white to dark gray, and are slightly sunken in lesions. They contain setose acervuli. Two isolates (CQ1, CQ2) were cultured in V8 media for the subsequent examination for their morphological characteristics and DNA barcoding [154].

CQ1 mycelia were gray, sparse and flat. They produced abundant sclerotia and conidia. The conidia were cylindrical, hyaline, and aseptate. The size of 50 conidia averaged 21 × 4.3 µm. CQ2 mycelia were gray to dark and fluffy. They produced abundant sclerotia, acervuli, and conidia. The conidia were falcate, hyaline, and aseptate. The size, averaged from 50 conidia, was 26.8 × 2.4 µm [154]. Both isolates have been identified by multigene sequencing, and the multiple sequence alignment of vouchered CBS isolates generated a maximum likelihood phylogenetic tree. Based on this information, CQ1 was identified as *Colletotrichum nigrum* and CQ2 as *Colletotrichum truncatum*. The sequences’ GenBank vouchers are: MN581860, MK675238, MF682518, and MK118057 [154].

For the completion of Koch postulates, 40-day-old quinoa plants (PI 634920) were inoculated on stems and leaves. Two weeks later, the stems showed bleached to tan sunken areas on wounded sites. After an extra week under humid conditions, the plants inoculated with *C. nigrum* produced acervuli (asexual stage) and sclerotia, whereas *C. truncatum* produced only acervuli. Infected stems were cultured in artificial media. The morphological characteristics of grown mycelia matched those of the initial inoculum used on the plants. Inoculated detached leaves developed brownish, circular lesions. This disease may cause lodging and emerge in new quinoa production areas, resulting in yield losses [154].

### 3.2. Fungi Identified by Morphological Approaches

#### 3.2.1. Brown Stalk Rot

Brown stalk rot was observed in *C. quinoa* growing in rotation with potatoes in the highlands of Puno, Peru, in 1974 and 1975. The organism was isolated from diseased stems of *C. quinoa* bearing pycnidia. As a practical first step for identification, the alkaline substance 1 *M* NaOH was added dropwise. Its purpose was to demonstrate the presence of substance “E” (a colorless metabolite from exigua) in malt extract agar cultures of the fungus to distinguish it from *Phoma exigua* var. *exigua* [155]. The test gave a positive result for *P. exigua* var. *foveata*, and comparative morphological characteristics with the causal agent of potato gangrene were carried out [156]. As both were similar and pathogenicity tests on potatoes were positive, the quinoa brown stalk rot’s causal agent was identified as *Phoma exigua* var. *foveata* (Foister) Boerema. Furthermore, isolates were sent to the Dutch Protection Service and the Commonwealth Mycological Institute (UK) for final confirmation [157].

**Figure 17:**
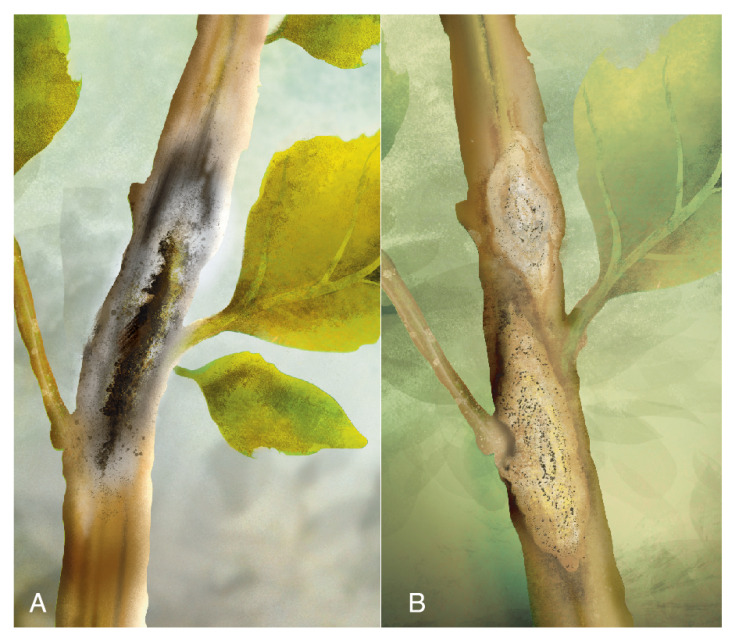
(**A**) Brown stalk rot. (**B**) Diamond-shaped symptoms bearing pycnidia. Source: illustrations adapted from Alandia et al. [4].

Symptoms were described as follows: small lesions on the higher third of the stem progress until reaching the upper part. At this stage, pycnidia are visible, the foliage wilts, the panicle does not form grain, and the brown stalk is prone to break (Figure 17A). The pycnidia are globose and dark brown; their size ranges between 101−116 µm in diameter. The ostiole is 30 µm in diameter, and the pycnidiospores are hyaline, ellipsoidal, unicellular, and biguttulated (small drop-shaped).

Their average size ranged between 6 × 2.2 µm in artificial media and 6.8 × 2.3 µm when coming from infected stems. Cross-inoculations, aided (and not aided) with mechanical wounds, were performed on potato plants and tubers, tomato plants, beetroot, sugar beets, and quinoa. Quinoa plants showed symptoms 3 dpi, potatoes and tomato plants showed foliar blight, potato tubers got black rot, whereas beetroot and sugar beets showed no symptoms. Overall, mechanical wounds increased the rate of infection, but pycnidia were rarely observed. The disease developed better at 3–5 °C than at 15–20 °C [157].

Based on in vitro experiments, it was hypothesized [157] that the highlands of South America are the geographic origin for the potato gangrene fungus *Phoma exigua* var. *foveata* because it is as pathogenic to potatoes as the virulent European isolates. However, on *C. quinoa* and *C. album*, it was more pathogenic. After inoculation, it caused a brown discolored area of rotting tissue, 1−3 cm long on both hosts, four dpi. On older leaves of both *Chenopodium* spp., concentric leaf spots of 0.5−1.0 cm in diameter were visible. The European strain caused similar spots, but one week later [158].

#### 3.2.2. Quinoa Diamond Black Stem/“Mancha Ojival del Allo”

Diamond black stem was observed in *C. quinoa* in the highlands of Puno, Peru, in 1974 and 1975. The disease is primarily present in the stem, with diamond-shaped lesions (2−3 cm), whitish to gray in the center, with brown edges and a vitreous halo. They bear pycnidia. At a later stage, the lesions join around the stem, causing it to collapse [133] (Figure 17B).

#### 3.2.3. Sclerotium in Quinoa

Stem rot affecting quinoa plants was observed at the Experimental Station of Kayr’a (Cuzco, Peru) during 1997. The mycelium was cultured, and pathogenicity tests were carried out on three-month-old plants of quinoa, amaranth, potato, frejol, sunflower, and *Lupinus mutabilis*. All plants were infected, and quinoa was the most susceptible. Morphological comparison with *Sclerotinia* from potatoes allowed the morphological identification of *Sclerotinia* sp., currently known as *Whetzelinia sp*. Inoculation with ascospores was followed by mycelial growth after 17 days. Dark sclerotia measuring between 4−9 mm appeared five dpi in PDA cultured at 10 °C. Apothecia developed 53 dpi at 16 °C and 12 days later produced ascospores. The fungi caused dry rot in the stem’s neck in quinoa, leaves wilted, and the disease moved towards the panicle [159].

#### 3.2.4. Damping-Off

**Figure 18:**
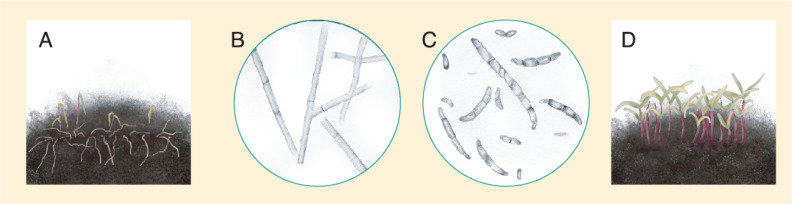
(**A**) Quinoa seedlings affected by damping-off. (**B**) *Rhizoctonia* spp hyphae. (**C**) *Fusarium* spp. spores. (**D**) Healthy quinoa seedlings growing under low soil moisture conditions. Source: illustration adapted from Isobe et al. (2019) [155].

1. Sensitivity of *Pythium zingiberum* and *P. butleri* oospores:
  Soil inoculation of oospores of *P. zingiberum* and *P.butleri* on soil caused damping-off of susceptible *C. quinoa* seedlings after ten days of incubation at 30 °C [160].
2. Seedling damping-off caused by Fusarium avenacearum and Pytium aphanidermatum:
  The fungi were isolated from infected stems of quinoa seedlings grown in a greenhouse. Microbes were morphologically described and the cultured fungi were inoculated on *C. quinoa* cv. Cochabamba. Pathogenicity tests confirmed that *P. aphanidermatum* and *F. avenaceum* were the causal agents of the damping-off of quinoa seedlings under greenhouse conditions. The seedling infection was significantly higher up to the first pair of leaves, showing that quinoa is most susceptible to the pathogens before emergence. However, the sum of post-emergence damping-off was significantly lower than that observed in sugar beets and higher than that observed in cabbage plants, except for *F. avenacearum*, which also produced marked susceptibility at the first true leaves stage. In addition to the two pathogens, *Ascochyta caulina*, *Fusarium spp*., and *Alternaria spp* were also isolated from infected tissue but could not infect quinoa seedlings during pathogenicity tests [139].
3. Pathogenicity tests on seedlings infected by *Rhizoctonia solani* and *Fusarium* spp.
  *Rhizoctonia solani* was isolated from the field in Peru. Pathogenicity tests performed in a greenhouse showed that *R. solani* prevented seed germination. It also created sunken lesions on the stems of old plants at ground level. *Fusarium spp*. reproduced wilting in old plants [4,161]. Quinoa seedling damping-off (Figure 18A) was observed during field experiments conducted at the experimental station of Nihon (Japan). It occurred from emergence until the four-leaf stage and increased under high soil moisture conditions. *Rhizoctonia* spp. (Figure 18B) and *Fusarium* spp. (Figure 18C) were identified morphologically from the symptomatic lesions [162].
4. Pathogenicity tests on seedlings caused by *Sclerotium rolfsii* Sacc
  *Sclerotium rolfsii* was isolated from diseased seedlings of *C. quinoa* in a field of Southern California. The susceptibility of *C. quinoa* to *S. rolfsii* was demonstrated in vitro and under greenhouse conditions [163].

## 4. Chemical Control for Oomycetes and Fungi

The control of oomycete and fungal diseases continues to rely mainly upon chemical measures for conventional agriculture. Fungicides can be effective only on a few closely related pathogens, in which case they are designated as narrow -spectrum fungicides, are often systemic, and usually have a single-site activity. In contrast, broad-spectrum fungicides can control a wide range of unrelated pathogens. In the case of oomycetes, 16 chemicals with different modes of action, translocations in the plant, types of activity, and risks of developing resistance, are available. A summary is presented in Table 7. However, sexual recombination, resulting in high pathogenic genetic diversity, as well as the migration rate, including low dispersal (within a few meters), increases local epidemics and the appearance of new pathogen genotypes in local populations. These facts will continuously affect sensitivity to fungicides, requiring the repeated adaptation of control strategies [105]. Several different target-site fungicides can achieve the chemical control of fungal pathogens in a mixture or in an alternating regime on the same crop. The most recent fungicides of this type are phenyl-pyrroles (P.P. fungicides) and dimethylation inhibitors (DMIs). They are considered the most effective chemicals registered to control diseases caused by *Ascomycetes* [164,165,166], depicted in blue on Table 7. The table aims to provide a general reference. The choice of fungicide is highly dependent on the availability and conditions of the particular fields to be treated.

## 5. Bacteria

### 5.1. Bacterial Leaf Spot Caused by Pseudomonas spp.

Bacterial leaf symptoms are small irregular spots both in leaves and stems. In leaves, they turn dark brown with concentric rings and a wet halo; in stems, they become necrotic, causing a deep lesion and wilting [133].

### 5.2. Bacterial Leaf Spot Caused by Pseudomonas syringae

**Figure 19:**
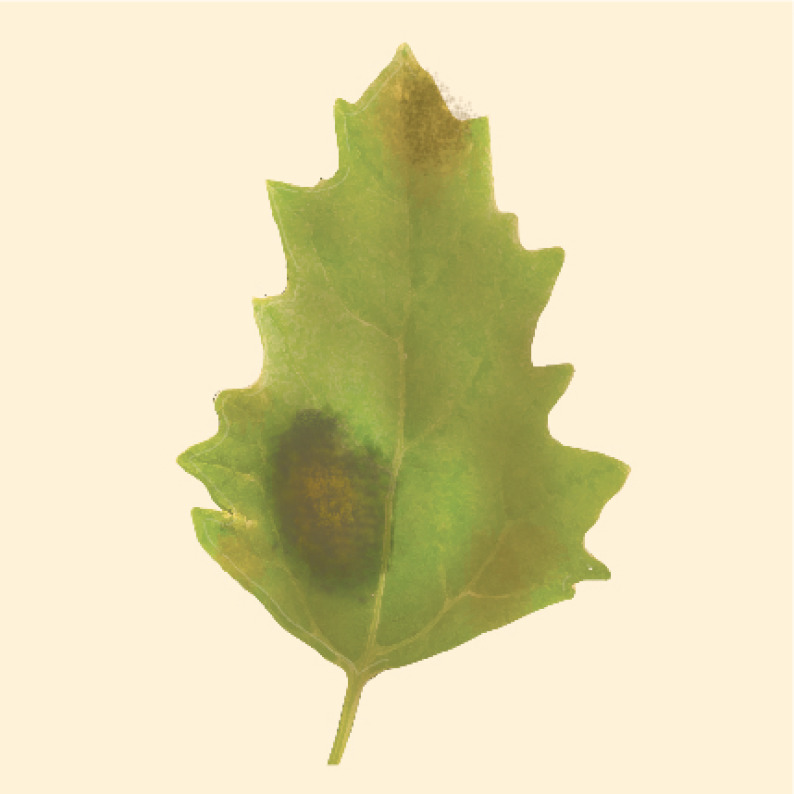
Symptoms of bacterial leaf spot on quinoa. Source: illustration adapted from Fonseca-Guerra et al. [167].

Bacteria were isolated from symptomatic leaves and inoculates on surface sterilized leaves of quinoa cv. Piartal. Between three to fice dpi, leaf spots were visible (Figure 19). The bacteria colonies were identified at the species level via morphology and molecula tools using a Bruker Daltonik MALDI Biotyper system (Germany). The coucher for the identified bacteria was uploades to the NCBI database as txid317 [167].

## 6. Viruses

Pathogenicity assays for the identification of viruses under greenhouse conditions require indicator plants. These plants show distinctive and consistent reactions to virus infections. Many plant viruses can be transmitted to indicator plants via mechanical infection or insects. *Nicotiana* (tobacco) and *Chenopodium* are hosts for a great number of viruses [168]. Therefore, *C. quinoa* could be infected with the viruses that infect host plants that grow next to it.

1. Chenopodium mosaic virus: Seedlings of *C. quinoa* were found to contain a highly infectious, seed-borne virus that may remain latent. The virus was restricted to the *Chenopodiaceae* and was similar to the soybean mosaic virus in morphology and physio-chemical properties [169].
2. Amaranthus leaf mottle virus (ALMV): Successful infections were achieved on *C. quinoa*, which exhibited chlorotic local lesions and severe systemic mosaic, leaf deformation, wilting, stunning, and finally collapse of the plants. Transmission via *Aphis gossypii* was confirmed 2 to 3 weeks after the 1-day inoculation access period [170].
3. Arracacha virus A: AVA is common in arracacha (*Arracacia xanthorrhiza*) in the region of the Peruvian Andes. AVA was not transmitted by *Myzus persicae*, but was transmitted by the inoculation of sap and is best propagated in *C. quinoa* and *Nicotiana clevelandii* [171,172,173].
4. Ullucus virus C: UVC is a comovirus prevalent in *Ullucus tuberosus* grown at high altitudes in the Bolivian and Peruvian Andes. It was transmitted mechanically to *C. amaranticolor* and *C. quinoa*. It caused a systemic infection. UVC was not transmitted by either aphid species (*Aphis gossypii* or *Myzus persicae*) or through seeds of *C. quinoa*. However, it was transmitted through leaf contact between infected and healthy plants, causing chlorosis [173].
5. Potato virus S (PVS): *Chenopodium quinoa* plants displayed symptoms of PVS infection 14 days after artificial inoculation with PVS [174,175].
6. Potato Andean latent virus: APLV was found to infect both *C. quinoa* and *C. amaranticolor* [176].
7. Cucumber mosaic virus (CMV): Partially purified extracts from leaves of *Phytolacca americana* caused marked inhibition of CMV infection on *C. quinoa* [177].
8. Tobacco mosaic virus: TMV has successfully infected *C. quinoa* [178].
9. Passiflora latent virus (PLV): *Chenopodium quinoa* plants presenting systemic symptoms after inoculation with PLV showed high concentrations of virus particles in their cytoplasm, mitochondria, and chloroplasts [179]
10. Plantago asiatica mosaic virus (PIAMV): Mechanical inoculation with infected sap of *Lilium* leaves on *C. quinoa* yielded chlorotic or necrotic local lesions [180].
11. Carnation latent virus: *C. quinoa* is an indicator species for the carnation latent virus [181].
12. Chlorotic leaf spot virus: Sap inoculation on *C. quinoa* resulted in a satisfactory infection [182].

## 7. Conclusions and Future Directions

The growing interest in quinoa has prompted research on all aspects of this crop. From the perspective of phytopathology, it is essential to collaborate as quinoa cultivation has been introduced to many countries worldwide and continues to enter new regions. Therefore, it faces different challenges in each area. The impact on final seed yield has not been quantified for many diseases yet, as they have only been identified causing symptoms on plant tissue, but it is essential to turn our attention to this aspect.

Determining the mycobiota in quinoa grain food is of prime importance. The presence of seed pathogens associated with mycotoxins is concerning. These secondary metabolites are generally produced by fungi belonging to the genera (*Alternaria*, *Aspergillus*, *Fusarium* and *Penicillium)* [183,184]. The latter two pathogens from this list have been isolated from quinoa plants. Thus, mycotoxin production may occur in the field or during post-harvest, storage, or processing [185]. Indeed, a recent comparative study [183] of mycotoxin occurrence in quinoa grains cultivated in South America, and North Europe found a large array of mycotoxins on Northern European grain. Mycotoxins were predominantly associated with *Fusarium* spp. (e.g., butenolid, aurofusarin, equisetin, culmorin), *Alternaria* spp. (e.g., tenuazonic acid and altersetin), *Cladosporium* spp. (e.g., Cladosporim), and *Penicillium* spp. (e.g., ochratoxin A, flavogaucin, and mycophenolic acid). Unspecific metabolites were also found in modest amounts. Cleaning seeds provided a considerable reduction (ca. 50%) in the content of mycotoxins, but overall the North European grains had considerably more mycotoxins compared to South American grains even after cleaning. Weather conditions, cultivation method and post-harvest treatments could explain mycotoxins array presence differences on grain examined. The resilience of Andean grains to the growth of mycotoxin-producing fungi could be due to their adaptation to their natural centre of origin. Something that drastically changes when quinoa is cultivated in other latitudes [186,187]. It could also be argued that high saponin-containing quinoa grains may prevent the growth of fungi [188] or serve as a fungistatic. Therefore, monitoring seed quality during post-harvest should become a routine procedure. The implementation of this practice will highlight the fragility of organic quinoa production in new temperate environments.

It is essential to standardize the descriptions of diseases, taking into account the following suggestions:

- Morphological identification paired with molecular tools for accurate descriptions of causal agents, published in scientific journals, as well as the sharing of knowledge within quinoa networks and conferences.
- The performance of inclusive pathogenicity tests and Koch’s postulates to clarify the type of interaction observed (e.g., pathogenic, endophytic/symbiotic, or saprophytic.).
- Standardized protocols for disease propagation and assessment methods for severity after infection.
- The development of strategies for seed sanitation.
- There exist several research centers located in areas where quinoa is traditionally grown, and recently a pilot global collaborative network on quinoa (GCN-Quinoa) (www.gcn-quinoa.org, accessed on 10 June 2021) has been established [189]. These networks primarily share knowledge on cultivation and plant breeding. Knowledge sharing in relation to quinoa diseases should also be considered.
- More research on methodologies for the rapid, high throughput screening of quinoa seeds and plants for the presence of economically important pathogens of quinoa is needed. This would be useful for detecting causal agents early in disease development and ensuring certified pathogen-free quinoa seeds. Moreover, phone apps with deep learning models for diagnosing various plant diseases and pest attacks are becoming interesting tools, which may be useful in the future.

## Figures and Tables

**Table 2:** Documented reports for downy mildew on *C. quinoa* and weedy *Chenopods.*

Country	*C. quinoa* Leaves (√), Seed (x)		*C.album* Leaves		*C. berlandieri* var. Macrocalycium		*C. murale* Leaves		*C. ambrosoides* Leaves		*C. ficifolium* Leaves		Researcher	Year	[Ref]
	Mor.	Mol.	Mor.	Mol.	Mor.	Mol.	Mor.	Mol.	Mor.	Mol.	Mor.	Mol.			
Bolivia	√												Martin Cardenas	1941	[36]
Peru	√												G. Garcia	1947	[37]
Canada	√												JF.Tewari	1990	[38]
Peru	√		√				√		√				L.Aragon	1992	[39]
Ecuador	√												Jose Ochoa	1999	[40]
Denmark	√												S. Danielsen	2002	[41]
Poland	√												Panka	2004	[42]
India	√												A. Kumar	2006	[43]
Bolivia	√	√											Erica Swenson	2006	[44]
Argentina			√	√									Y.J. Choi	2008	[26]
China			√	√										2010	[29]
Ireland			√	√											
South Korea			√	√											
Netherlands			√	√											
Germany			√	√											
Latvia			√	√											
Romania			√	√											
Italy			√	√											
Peru		√											S. Danielsen	2010	[30]
Ecuador		√													
Denmark		√													
India							√						P. Baisvar	2010	[45]
USA (Pennsylvania)	√	√											Ana Testen	2012	[46]
Bolivia		x											Ana Testen	2014	[32]
Ecuador		x													
USA		x													
Korea	√	√											Y.J. Choi	2014	[47]
Morocco	-	-											Manal Mhada	2014	[48]
Egypt	√	√											Walaa Khalifa	2018	[49]
USA (N. Hampshire)	√	√	√	√	x√*	x√*						√**	Helen Nolen	2019	[50]
Turkey			√	√									M.Kara	2020	[31]
Turkey	√	√											Esra Gül	2021	[51]
Denmark	√	√	√	√									C. Colque-Little	2021	[52]

Mor. = morphological characterization; Mol. = molecular identification. Source: elaborated from references on the column [Ref]. x√* Koch postulates failed; √** corresponds to a field population.

**Table 3:** *Peronospora variabilis* sporangium sizes when isolated from *C. quinoa* and *C. album.*

*P. variabilis* Sporangium Isolation Origin		
*C. quinoa*	*C. album*	
av. Length × Width (µm)	av. Length × Width (µm)	Reference
25.5 × 17.5		Khalifa and Thabet 2018 [49]
22 × 23.13		Yin et al., 2018 [72]
27.5 × 20		Gül, 2021b [51]
28.8 × 21.8		Danielsen & Ames, 2004 [54]
30.7 × 23.8		Choi et al., 2010 [29]
31 × 23		Testen et al., 2012 [46]
28.5 × 23.5		Choi et al., 2014 [47]
	29.5 × 23	Choi et al., 2008 [26]
	30 × 25	Kara et al., 2020 [31]
27.7 × 21.0	30.1 × 24	av. size
1.32	1.25	av.ratio

**Table 4:** Set of quinoa cultivars and *Chenopodium album* postulated for profiling the virulence of *Peronospora variabilis.*

Cultivar	Hypothesized Resistance Factors	Response to Downy Mildew		Origin
		% Severity	% Sporulation	
* *C. album* *	R1, R2, R3, R4	5	0.04	Denmark
Puno	R1, R2, R3, R4	11	0.2	Denmark
Rosa Blanca	R1, R2, R3	32	17	Bolivia
Blanca	R1, R2, R3	46	47	Bolivia
Titicaca	R1, R2	52	40	Denmark
Vikinga	R1	70	69	Denmark

**Table 5:** Phenotypic infection traits and Ratio for representative cultivars and reference varieties.

Name	% Severity	% Sporulation	Spo/Sev Ratio	% Incidence	Ratio Based Classification
*C. album*	5	0.4	0.08	45	Resistant
*Puno*	11	0.2	0.02	42	Resistant
*Cv6 (Rosa Blanca)*	32	17	0.53	59	Highly tolerant
*Cv17 (Canchis)*	41	30	0.73	73	Mildly resistant
*Cv18 (Pandela Roja)*	45	29	0.64	74	Mildly resistant
*Cv16 (Kurmi)*	45	50	1.1	56	Susceptible
*Blanca*	46	47	1	79	Mildly susceptible
*Cv8 (Blanquita)*	50	69	1.4	67	Very susceptible
*Titicaca*	52	40	0.77	81	Mildly resistant
*Cv3 (Ayrampu)*	52	63	1.2	77	Susceptible
*Cv20 (Aynoka)*	58	63	1.1	83	Susceptible
*Cv21 (Mariqueña)*	71	84	1.2	82	Susceptible

**Table 6:** Eco-regions for quinoa production in South America.

				Temperature		
Eco-Region	Soil	Altitude m.a.s.l	Rainfall (mm)	Max.	Min.	Av.
Northern Highland shores of Lake Titicaca	Rich in organic matter	3500–4000	500	14	4	7
Central Highland	Slightly acid	3300–4100	350	17.7	−2	8.7
Southern Highland	Arid and poor soils	3200–4000	50–200	18	−11	5.7
Andean Slopes	Variable	800–3200	3500–700	12	3	7.6
Coastal/Lowland Northern, Central, and Southern	Variable	Sea level to Mountain range	40 > 2000	23 21 17	−8 7 6	4.5 14 11

Within the sub-regions, temperatures vary depending on location (coast or foothills), not shown. Source: elaborated with information from Gandarillas et al. (2015) [112]; Seiler et al. (2013) [120]; Cereceda et al. (2008) [121]; http://germoplasma.iniaf.gob.bo (accessed on 15 April 2020).

**Table 7:** Major fungicide groups and key active ingredients, application site, and resistance risk. Adapted from Gisi and Zierotski (2015) [105]; Lebeda and Cohen (2021) [165]; Plimmer, (2003) [166]; and Masielo et al., (2019) [164]. Rows in blue correspond to fungicides that are effective against *Ascomycetes.*

Mode of Trans-location	Fungicide Group and Key Active Ingredients	Resistance Risk ^a^	Foliar	Seed	Soil	Type of Activity	Translocation in Plants	Biochemical Mode of Action
Fully Systemic	Phenylamides: Metalaxyl, mefenoxam, oxadixyl, benalaxyl, kiralaxyl, ofurace	High	√	√		Preventive, curative, eradicative	Apoplastic, symplastic, translaminar	Inhibition of rRNA synthesis
Partially Systemic	^b^ Quinone outside inhibitors: Azoxystrobin, fenamidone, famox, adone, trifloxystrobin: kresoxin-methyl, Pyraclostrobin		√	√		Preventive	translaminar apoplastic	Inhibition of mitochondrial respiration at enzyme complex III
Non-Systemic	^b^ Multisites: For example, mancozeb; chlorothalonil, copper, cu-oxychloride, cu-hydroxide; folpet; thiram, chlorothalonil	Low	√			Preventive		Multi-site inhibition
Non-Systemic	Carboxylic acid amides: Dimethomorph, flumorph; iprovalicarb, benthiavalicarb; mandipropamid	Moderate	√			Preventive	Translaminar	Cell wall synthesis, Ces3A cellulose synthase inhibition
Fully Systemic	Cyanoacetamide, oximes (cymoxanil)	Moderate	√	√		Preventive, curative	Apoplastic, symplastic, translaminar	Inhibition of mitochondrial respiration at the enzyme complex III
Non-Systemic	Dinitroanilines (fluazinam)	Moderate				Preventive		Inhibition of ATP production
Fully Systemic	Phosphonates (fosetyl-Al)	Moderate	√			Preventive, curative	Apoplastic, symplastic,	Inhibition of spore germination, retardation of mycelia
Partially Systemic	Quinone inside respiration inhibitors: Cyazofamid, amisulbrom	Medium to hight	√	√		Preventive, curative, eradicative/	Translaminar	
Fully Systemic	Benzamides (fluopicolide)	Mod.	√		√	Preventive, curative	Apoplastic, symplastic, translaminar	Delocalization of spectrin-like proteins
	Benzamides, carboxamides Ethaboxam, zoxamide	Low						
Systemic	Hymexaxol (heteroaromatics)			√	√			Fungal RNA and DNA syntheses
Contact	^b^ Thiadiazoles ( Etridiazole)				√	Preventive, curative		Lipid structure of Mitochondria
Resistance inducer	Acibenzolar-S-methyl.			√				
	^b ^ Demethylation inhibitor fungicides (DMIs): Imidazoles, triazolinthiones, triazoles prothioconazole, prochloraz, terbuconazole, difenoconazole		√	√		Preventive, curative		Sterole biosynthesis in membranes
	^b ^PP fungicides (Phenylpyrroles) phenylpyrroles Fludioxonil		√	√		Preventive, curative		Signal transduction
Fully Systemic	Carbamates: Propamocarb, prothiocarb					Preventive, eradicative	Apoplastic	Multi-site inhibition Affecting the membrane

**^a^** Nomenclature according to Fungicide Resistance Action Committee mode of action code list, 2014, www.frac.info (accessed on 10 June 2021). **^b^** Quinone outside inhibitors and multi-sites are broad-spectrum fungicides, including activity against fungi.

## Data Availability

Not applicable.
